# Australian and New Zealand Fish Oil Products in 2016 Meet Label Omega-3 Claims and Are Not Oxidized

**DOI:** 10.3390/nu8110703

**Published:** 2016-11-05

**Authors:** Peter D. Nichols, Lalen Dogan, Andrew Sinclair

**Affiliations:** 1 CSIRO Oceans and Atmosphere, GPO Box 1538, Hobart TAS 7000, Australia; 2 DSM Nutritional Products Asia Pacific, 30 Pasir Panjang Road, Mapletree Business City, #13-31, Singapore 117440, Singapore; lalen.dogan@dsm.com; 3 School of Medicine, Deakin University, Geelong, VIC 3220, Australia; andrew.sinclair@deakin.edu.au; 4 Department of Nutrition & Dietetics, Monash University, Clayton, VIC 3800, Australia

**Keywords:** *n*-3 LC-PUFA, EPA, DHA, peroxide value, Australian and New Zealand fish oils

## Abstract

We provide new fish oil product results to assist industry in Australia and New Zealand and, ultimately, consumers in understanding the high product quality assurance protocols in place, together with the high product quality that has been determined by both industry and independent laboratories. Fish oil capsule products common to Australia and New Zealand were purchased in May 2016 in Richmond, Victoria, Australia. Products were from two groups; five standard fish oil products and five fish oil concentrates. Noting Therapeutic Goods Administration (TGA) requirement for use of standard methods, for all analyses undertaken a laboratory was selected that met the TGA criteria, including with accreditation. Total *n*-3 content exceeded the label-claimed content for all 10 products, with supplements containing on average 124% of the claimed content (range 115%–136%); eicosapentaenoic acid and docosahexaenoic acid (EPA + DHA) content averaged 109% of the label claim (range 99%–119%). All 10 products (100%) similarly met the international recommended peroxide value (PV) level. Anisidine value (pAV) met the international recommended level for eight of the 10 products, with two products known to contain flavorings that interfere with the pAV test. When accredited laboratories and standard protocols are used, Australian and New Zealand fish oil products have been shown to clearly meet their label claims for EPA + DHA content, and are not oxidized.

## 1. Introduction

The health benefits of omega-3 long-chain (≥C_20_) polyunsaturated fatty acids (LC-PUFA, also termed LC omega-3 or more generally *n*-3 oils by some user groups) were first documented over three decades ago. Danish researchers reported that Greenland Eskimos had lower incidence of heart disease than other ethnic groups in spite of their high fat diet that was rich in marine mammal blubber [1]. The main LC omega-3 oils attributed to cause this health benefit were eicosapentaenoic acid (EPA, 20:5*n*-3) and docosahexaenoic acid (DHA, 22:6*n*-3). For simplicity, we use the term LC omega-3 in this paper in consideration of both fatty acids, and also that of docosapentaenoic acid (DPA, 22:5*n*-3), which is also a minor component of fish and fish oils. The traditional main source for these health-benefitting LC omega-3 oils has been seafood [2]. Since the early research, in excess of 30,000 scientific papers have been published examining the health benefits of LC omega-3 oils, with around 80% of the studies showing numerous health benefits are associated with consumption of these key nutrients [3]. Of the remaining studies, most of these do not demonstrate clear benefits, and this is generally attributed to the complexity of nutritional research and other factors [4], while only a few show negative results.

Against the large and clear body of evidence supporting the benefits of and the need to increase consumption of the health-benefitting LC omega-3 oils, particularly for those on largely western diets, several papers have been published in early 2015 [5] and again in mid-2016 [6] reporting that New Zealand (and Australian) products contained substantially lower EPA + DHA content than label claims and were also heavily oxidized. The 2015 results [5] were not obtained from an accredited laboratory, and nor were standard protocols used [7,8]. The 2015 study [5] specifically stated that ‘fish oil supplements in New Zealand are highly oxidised and do not meet label content of *n*-3 PUFA’. The second New Zealand study was conducted using laboratory oxidized fish oil [6], with the justification being the results of the early 2015 paper [5]. The authors investigated both percentage input of *n*-3 EPA + DHA content (measured *n*-3 content expressed as a percentage of the label claim) and oxidation levels of fish oil supplements purchased in retail outlets in New Zealand. The selected products were not identified by brand name, but they came from both Australian and New Zealand companies. Media in the region and elsewhere picked up on 2015 paper [5] and television, newspaper and radio reports were particularly negative in New Zealand.

In this study, we report new results and other information to assist the Australian and New Zealand industry and ultimately consumers and other end users in understanding the high product quality assurance protocols already in place, together with the high product quality that has been determined by both industry and independent laboratories.

## 2. Materials and Methods

Fish oil capsule products were purchased in May 2016 from the Chemist Warehouse outlet in Bridge Road, Richmond, Victoria. Products were from two groups; five were standard fish oil products and five were fish oil concentrates (Table 1). The brands are common to Australian and New Zealand outlets. Two bottles were purchased of each brand, with one bottle archived for possible follow up work. For nine of the ten products, the two bottles were from identical batches. For one product only, the two bottles were from separate batches.

Sample containers were not opened and were taken directly by one author from the site of purchase to ALS Food & Pharmaceutical, located at Scoresby, Victoria. The samples were then transported to the ALS Rydalmere, NSW facilities for the analyses, with analyses performed blind. The ALS Rydalmere facilities maintain a GMP license for analysis held with the TGA and Australian Pesticides and Veterinary Medicines Authority. Samples were analysed within 10 days from purchase, with reporting on peroxide value (PV), anisidine value (pAV) and mg/g fatty acids (FA) occurring in June and for the percent FA in September 2016. Details on the 10 products analysed are shown in Table 1.

Full details on analytical methods are available on request from ALS; briefly, British Pharmacopoeia (BP) methods were used for PV and pAV determinations, with ALS method P111 used for fatty acid analyses. P111 is an ALS in house method, based on BP 2.4.22 with slight modifications to the column length and the addition of another internal standard. Oil pooled from 20 capsules per Brand was used for the three analyses—PV, pAV, FA. For the sample preparation for FA determinations, the derivatisation procedure involved simple breakdown of diluted oil, with sodium hydroxide in methanol added and heating for 30 min, then cooling, followed by addition of boron trifluoride. After heating, cooling, addition of hexane and mixing, then addition of saturated sodium chloride solution and shaking, the layers were allowed to separate; the top layer was then taken and analysed by gas chromatography (GC). The concentration and the percentage level of FA in the fats and oil samples was determined using an Agilent 6890 GC fitted with a polar column, split injector and flame ionisation detector. For the EPA + DHA determination, an appropriate amount of sample was dissolved in 1 mL internal standard (containing heptadecanoic acid and lauric acid). Samples were analysed in the same run alongside a standard preparation with known concentrations of EPA and DHA. All analyses (PV, pAV, FA) were performed in duplicate, with the data presented here being the mean values.

## 3. Results

### 3.1. EPA + DHA Content

**Figure 1:**
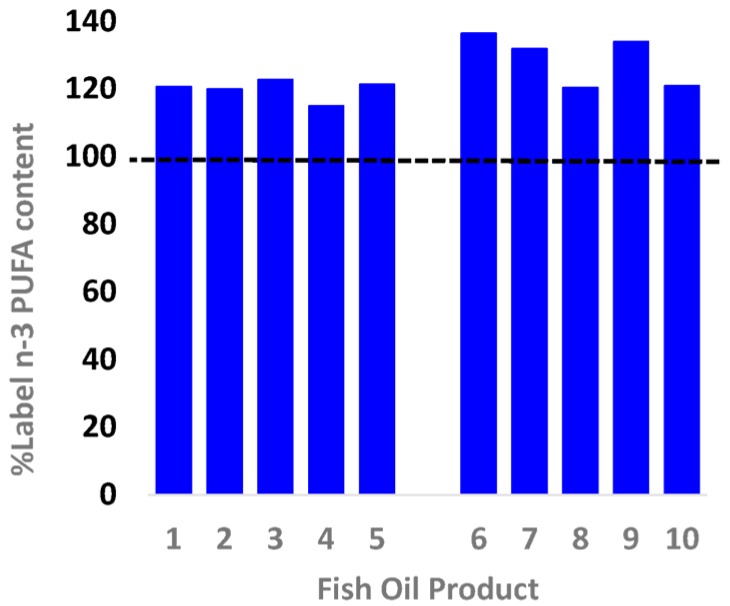
The actual *n*-3 polyunsaturated fatty acid (PUFA) content contained in individual retail fish oil brands in relation to the content claim (dotted line). Both values (label and actual) are shown in Table 1. The format used is the same as [5].

The 10 products were analysed for total *n*-3, including EPA + DHA content (Figure 1). The total *n*-3 content exceeded the label-claimed content for all 10 products, with supplements containing on average 124% of the claimed content (range 115%–136%, Figure 1). On average, EPA + DHA was 109% of the claimed content (range 99%–119%). The standard fish oil products (1–5) varied in terms of cost, number of capsules in the container, with product 5 also containing greater amounts of EPA + DHA per capsule; this was due to the size of the capsule (1500 mg) being greater than products 1–4 (all 1000 mg). Overall the cost to consumers to obtain the National Health and Medical Research Council (NHMRC) recommended intake (~500 mg/day of LC omega-3; 610 mg/day males, 430 mg/day females) via intake of the standard fish oil products varied twofold from 5 to 12 cents, or $15.50 to $45 (average $31) on a per annum basis.

The fish oil concentrate products (6–10) also showed variation in cost, the number and size of capsules, and thereby also the cost to consumers to obtain the recommended NHMRC intake (Table 1). The overall cost to consumers was higher than that for the standard fish oil products, at $33 to $103 (average $81) per annum.

The percent composition of the major fatty acids, including EPA, DPA and DHA, is presented in Table 2. The five standard fish oil products (1–5) showed similar profiles, including for the 3 LC omega-3 oils; the profiles were similar to those reported previously for such products [9,10]. The five concentrate products contained comparatively elevated levels of the three LC omega-3, with EPA + DHA in the range 59%–66% of total fatty acids (Table 2); once again these results were similar to enriched products previously reported [9,10]. EPA ranged from 36% to 41%, with DHA varying from 21% to 26%.

### 3.2. Oxidative Markers

**Figure 2:**
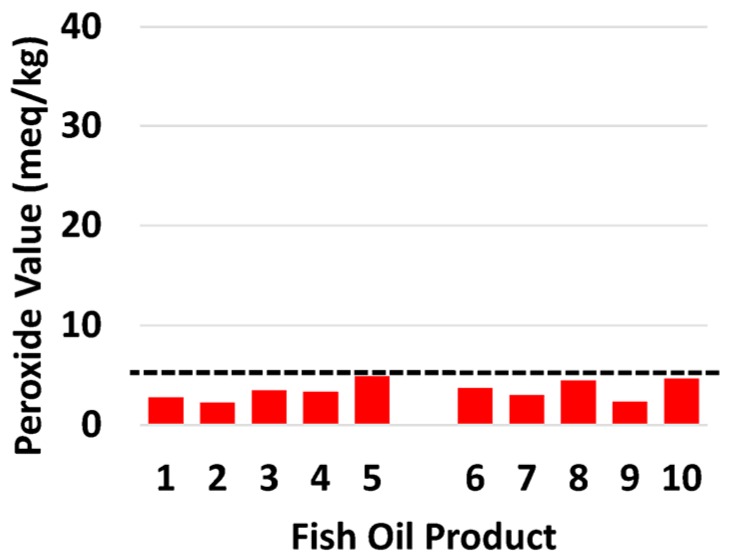
The content of the oxidation marker peroxide value in individual retail fish oil brands in relation to the recommended international threshold (dotted line; GOED Voluntary Monograph maximum limit—5 meq/kg. Australian government maximum limit—10 meq/kg). The format used is the same as [5].

Levels of oxidation of all products were assessed (Table 3). All 10 products (100%) met the international recommended PV level (Figure 2). Anisidine value met international recommended level, for 8 of the 10 products. Products 3 and 10 (pAV 108, 109 respectively) were known to contain flavorings that interfere with the pAV test [11].

## 4. Discussion

This paper builds on an initial article prepared by the Australian and New Zealand Omega-3 Centre [7,8]. LC omega-3 oil supplements, as complementary medicines, are regulated by the Therapeutic Goods Administration (TGA) in Australia and Medsafe (New Zealand). The two national regulators have quality Good Manufacturing Practice (GMP) standards in place that the industry has to adhere to, including long-term stability testing relative to a set quality standard for oxidation and also meeting label claims on EPA + DHA content. Both the industry and regulators use standard analytical methods. The manufacturers and brand owners for omega-3 supplements are required to keep records and meet these standards, so consumers should feel comfortable they are getting safe and reliable supplements. Noting the TGA requirement above for the use of standard methods, for all analyses presented here, a laboratory was selected that met the TGA criteria, including having accreditation.

Our new data reported in this study for a range of fish oil product brands available in Australia and New Zealand clearly demonstrates that all products examined meet the *n*-3 label content claims, and were not heavily oxidized. The results contrast with those of the 2015 University of Auckland study [5]. The Australian and New Zealand headquartered Omega-3 Centre and its sister association in the USA, the Global Organisation for EPA and DHA (GOED), along with the regional New Zealand Institute of Chemistry (NZIC) Oils and Fats Group and Australasian Section of the American Oil Chemists’ Society (AAOCS), consulted with a large number of lipid science and omega-3 experts who carefully examined the University of Auckland study. Those consulted were rather surprised by the New Zealand results, especially as the Australian government science agency, CSIRO and collaborators, had performed similar research published in 2014 on both percentage and also absolute content of EPA + DHA in a range of products purchased in Australia and available in New Zealand; these studies reported the samples were predominantly compliant with label claim [9,10], although these studies were not cited by the New Zealand authors. In addition, other analyses on the oils to confirm their generally high triacylglycerol (TAG) content were performed by the CSIRO researchers (data not shown); this finding of high TAG is consistent with the oil not being oxidized nor containing breakdown products. Interestingly, during 2015, a different team from the University of Auckland reported on the high oil quality for a newly developed fish oil product. This new fish oil was compliant to its stated label regarding its EPA + DHA content, and importantly it was not oxidized [12]. The University of Auckland papers [5,6] also did not refer to the co-occurring New Zealand study.

As follow up to the University of Auckland fish oils paper [5] and the resulting media attention, the TGA did a suite of new analyses, testing similar fish oil products on the Australian and New Zealand market. The TGA tested fifteen fish oil supplements using the prescribed methods of analysis for the industry [13,14] Eight products were ‘fish oil’ captured under the TGA Compositional Guideline for ‘fish oil—natural’. Seven products were captured under the British Pharmacopoeia (BP) monograph on ‘Concentrated Omega-3 triglycerides—fish’. The products were analysed using BP methods for testing fish oils. The TGA results were found to be acceptable for both oxidation status and for omega-3 fatty acid content. In terms of specific details, the testing for oxidation gave satisfactory results for all products in relation to peroxide value. Four products gave high results for anisidine value, which was attributed by the TGA to the presence of excipient fragrances or flavourings (aldehydes) which interfere with the test.

Our new results presented in this study showed similar outcomes to the TGA analyses; several products did show elevated pAV, with these results attributed to the presence of additives as stated on the labels and/or from consultation with the companies.

All 15 products analysed by the TGA gave acceptable results for their content of omega-3 fatty acids; that is, they were all above the legislated (or official) lower limit of 90% of label claim [13,14]. Industry had also performed re-testing of products which gave similar results to the TGA results; again the EPA + DHA were meeting label claims, and the products were not oxidized. As it was noted that non-standard procedures had been used in the University of Auckland study [5], the Omega-3 Centre contacted the authors soon after their publication was released to discuss the results and the test methods used. However, the authors stood by their results. Both the industry and a range of scientific experts and bodies, in particular the TGA, believed the data to be atypical of analyses conducted by accredited laboratories.

After consultation within the industry, and due to the unusual nature of the results for the 2015 University of Auckland study [5], GOED also set up a scientific testing program to conduct randomised testing of omega-3 fish oil supplements selected exclusively from the New Zealand market. GOED is currently finalising their fatty acid results, which have also generally shown compliance [15], and it intends to share them with the industry and the regulators. GOED recently published a review on the topic of oxidation of EPA and DHA rich oils [11]. For over 2000 published analyses of fish oil capsules included in the review, 2171/2187 products analysed were compliant for PV, with 2092/2117 products compliant for pAV, both with respect to Australian Government, British Pharmacopoeia and European Pharmacopeia standards. GOED also stated the use of TOTOX as a measure of oxidation (a calculation combining PV and pAV) was not valid for any oils containing other interfering ingredients (many oils are flavoured) or that have strong colours, including krill oils and virgin salmon oils [11]. In recognition of the methods issues that are recognized to occur, GOED and the Council for Responsible Nutrition (CRN) published and widely distributed a White paper [16] prior to the completion of the peer review scientific paper [11].

The GOED White paper aimed to improve researcher and industry knowledge on protocols for oxidation testing. The summary of the White paper states: Oxidation of omega-3 oils is a complicated topic, but it is important to understand. A significant number of consumers cite fishy burp and/or taste, thought to be the result of oxidation, as one of the main reasons they do not consume EPA and DHA oils. In addition, some papers have discussed the potential for adverse effects associated with consumption of oxidized oils. Measuring oxidation in omega-3 oils is complex from a chemistry point of view and in addition due to the differences in chemical and physical characteristics of many commercially available products, which means not all methods to determine quality are appropriate for all types of oils. A number of organizations and consumer groups issue seals for product quality seals, and academic groups have published data on levels of oxidation in omega-3 oils. Overall, these data show that commercially available omega-3 supplements are low in oxidation products [11]. The White paper prepared by GOED and the CRN responds to the methods issues widely recognized to have occurred in the New Zealand fish oils study and a further recent and similar Canadian study [17].

The University of Auckland fish oils study [5] highlights an issue seen in the industry and academia for many years: the lack of use of consistent testing methods across commercial and research laboratories. The American Oil Chemists Society (AOCS) and other industry associations including GOED provide support for science around rigorous and validated analytical testing of oils. Proficiency programs are offered by AOCS and several organizations.

In contrast to the publication and commentary on the two University of Auckland papers [5,6], it can be noted that further positive news is available. For example, the peer-reviewed journal Nutrients published a special issue in 2014. The issue is also available as a stand-alone book on Australian and New Zealand research on omega-3 oils. The book showcases a range of forward looking and also new and generally positive scientific outcomes occurring in the omega-3 field [18].

## 5. Conclusions

In conclusion, we emphasize that a range of analyses including those performed in this study, and in addition by the TGA, GOED and by other groups and industry have been performed on fish oil products available in Australia and New Zealand using validated protocols and including in accredited laboratories. All analyses confirm the high quality of the Australian and New Zealand fish oil products. The overriding message from our results, those of the TGA and others noted above, has been that the Australian and New Zealand fish oil products do meet omega-3 PUFA specification, and are not oxidized. The new Omega-3 Centre results are provided here for access and discussion by the wide range of concerned groups, societies, industry, and importantly the consumers seeking high quality Australian and New Zealand LC omega-3 containing fish oil products.

## Figures and Tables

**Table 1:** Fish oil capsules—brand and composition details as supplied by manufacturers, and as determined in this study.

Product	Sample	Batch	Number	EPA	DHA	Total	Capsule	*n*-3	% Label	Cost	Cost	Cost per	Cost per
	Number	Number	Capsules			*n*-3	Size	Content	*n*-3 PUFA	per	per	500 mg	Annum for
								Claim	Content	Bottle	Capsule	EPA + DHA	500 mg
													EPA + DHA
				mg/cap	mg/cap	mg/cap	mg	mg		$	$	$	$
Standard Fish Oil Triacylglycerols													
Blackmore Fish Oil 1000	1	275257	200	178	133	362	1000	300	121	15.99	0.08	0.11	40.35
Healthy Care Fish Oil 1000	2	677168	400	186	127	360	1000	300	120	12.99	0.03	0.05	16.48
Natures Own Fish Oil 1000	3	14530058	400	190	132	368	1000	300	123	17.99	0.04	0.06	22.30
Natures Way Fish Oil 1000	4	151967	200	176	122	345	1000	300	115	16.99	0.08	0.12	44.96
Swisse Wild Fish Oil 1500	5	52112A	200	279	193	546	1500	450	121	17.99	0.09	0.08	30.08
Fish Oil Concentrates													
Blackmores Omega Triple	6	276923	150	651	423	1228	1500	900	136	31.99	0.21	0.09	77.84
Bioglan Super Fish Oil	7	20674A	200	413	287	791	1000	600	132	17.99	0.09	0.06	32.83
Natures Own Triple Concentrated	8	1469561	70	566	392	1083	1500	900	120	16.99	0.24	0.11	88.59
Natures way Triple Strength	9	160061	60	522	379	1045	1500	780	134	16.99	0.28	0.14	103.36
Swisse 4x Strength Concentrate	10	160116	90	757	539	1450	1800	1200	121	24.99	0.28	0.10	101.35

Sample numbers refer to Figure 1 and Figure 2. Cost per annum for fish oil concentrates is based on the cost of one capsule.Abbreviations: EPA, eicosapentaenoic acid; DHA docosahexaenoic acid; PUFA, polyunsaturated fatty acids; cap, capsule.

**Table 2:** Fish oil capsules—fatty acid (FA) composition (as % of total FAs) determined in this study.

Product	1	2	3	4	5	6	7	8	9	10
Fatty acid										
14:0	7.5	6.7	7.6	6.4	6.7	0.4	0.3	0.4	0.1	0.4
15:0	0.5	0.6	0.5	0.5	0.5	0.0	0.0	0.0	0.0	0.0
16:0	15.7	15.3	15.4	15.6	14.5	0.5	0.5	1.0	0.2	0.6
16:1*n*-7c	8.7	8.2	9.5	7.8	8.5	0.3	0.3	0.5	0.3	0.3
16:3 + 16:4	1.3	1.1	1.3	1.1	1.3	0.0	0.0	0.0	0.0	0.0
17:0	0.5	0.5	0.5	0.5	0.4	0.0	0.0	0.0	0.0	0.0
18:0	3.1	3.0	3.0	3.3	3.1	3.0	2.4	4.6	1.8	3.7
18:1*n*-9c	8.5	9.9	7.6	8.1	9.8	3.4	2.9	7.2	3.1	4.5
18:1*n*-7c	3.1	3.0	3.1	3.0	3.4	1.5	1.3	2.7	1.3	1.7
18:2*n*-6	3.5	3.2	3.6	3.2	3.5	0.7	0.6	1.2	0.5	0.9
18:3*n*-3	1.0	0.9	0.9	0.9	1.1	1.2	1.0	1.3	0.8	1.2
18:3*n*-6	0.2	0.2	0.3	0.2	0.3	0.1	0.0	0.1	0.0	0.1
18:4*n*-3	2.9	2.5	2.6	3.1	3.0	0.8	0.8	1.7	0.7	0.9
20:1*n*-11c/*n*-9c	1.07	1.5	0.9	1.8	1.2	3.6	3.4	2.5	3.9	3.0
20:2*n*-6	0.4	0.4	0.3	0.4	0.4	0.7	0.6	0.6	0.5	0.6
20:3*n*-6	0.1	0.1	0.1	0.1	0.2	0.5	0.4	0.3	0.4	0.4
20:4*n*-6	1.1	1.4	1.1	1.4	1.2	0.1	0.1	0.1	0.3	0.1
20:4*n*-3	0.7	0.7	0.7	0.9	0.8	0.1	0.2	0.1	0.2	0.1
20:5*n*-3 (EPA)	17.5	18.1	18.4	17.6	18.4	40.5	39.3	35.9	36.2	39.2
21:5*n*-3	0.7	0.7	0.7	0.7	0.7	1.8	2.0	1.2	2.1	1.4
22:1*n*-11c/*n*-9c	0.52	1.2	0.5	1.2	0.6	0.6	2.0	1.3	4.5	1.6
22:5*n*-6 (DPA6)	0.1	0.0	0.5	0.1	0.0	0.2	0.0	0.2	0.0	0.0
22:5*n*-3 (DPA3)	1.9	1.8	2.0	1.9	1.9	3.4	4.6	3.1	5.2	3.2
22:6*n*-3 (DHA)	12.4	11.7	12.1	11.7	11.8	25.0	25.6	23.4	23.9	21.3

Product numbers refers to Table 1. EPA, eicosapentaenoic acid; DPA, docosapentaenoic acid; DHA, docosahexaenoic acid.

**Table 3:** Fish oil capsules—levels of oxidative markers determined in this study.

Product	1	2	3	4	5	6	7	8	9	10
PV (meq/kg)	2.77	2.24	3.45	3.32	4.87	3.68	2.97	4.45	2.3	4.63
pAV	13	10.4	108	25	29.9	20.2	4.44	14	109	6.23

PV denotes peroxide value; pAV denotes anisidine value. Product numbers refers to Table 1. GOED Voluntary Monograph maximum specifications: PV, 5.0 meq/kg; 20 pAV. Australian government and BP maximum specifications: PV, 10.0 meq/kg; 30 pAV.
